# Lost art of argumentation

**Published:** 2017-06-30

**Authors:** Jesse Leontowicz

**Affiliations:** 1 University of Saskatchewan, Saskatchewan, Canada

In training to be physicians, medical students occasionally try their hand at structured debates over popular topics in medicine. These debating exercises highlight an important issue for physicians: we may be known for our smarts, but we are not always known for our stellar communication skills. Much of our job is made easier by the implicit trust patients irrevocably afford us, but what happens when patients don’t implicitly trust us? We aren’t exactly great orators nor are we apt convincers. Most doctors will grimace when they tell you how they have failed to convince a mother to vaccinate her child or have a patient turn and be angry at them over a spoken misunderstanding. But is it a surprise that physicians are not great speakers? The typical medical student is motivated by knowing solid (mostly bio-medical) facts, you cannot argue the interpretation of an anterior ST segment elevation after all. But, ethics? The right to assisted-suicide? These high-stakes questions are the bread and butter of non-medical professions such as lawyers and journalists. Yet medical students actively suppress their opinions to provide non-judgemental care to the patient. By the nature of these debateable questions, one *needs* to allow for one’s personal self to influence the discussion, but medical students are wary of offending. Trying to disagree with a colleague can lead to some heated debates and bad blood… and who wants that in the clinic? Debating a controversial topic is not the forte of a medical student – that is the lesson I learned from the organized debates with students; and knowing your weakness is just half the battle.

Communication and the ability to debate is a critical skill for any leader, and physician leadership is a desperately needed role for Canada.^1^ While the idea of a physician leader sounds great on paper - few are effective leaders if we can be honest. Lawyers and businesspeople dominate the leadership ranks despite physicians having the longest training period. The need for communication and leadership training is evident to me after witnessing the particularly lackluster performance during the debates. I think it is critical that students such as myself take every opportunity at hand to improve communication and debating skills. The importance of verbal statements represent potentially powerful interventions for our patients, and words need to be chosen very carefully and respectfully. We must strive for finding a balance between our passionate personal views and relaying them with the kindness and compassion our professional is lauded for. Our curriculum designers should focus on these skills and provide opportunities to students by incorporating debate and leadership training into curricula. By improving our skills, we can be effective physician leaders for our communities, hospitals, and health regions.
